# Salvage Radioligand Therapy with Repeated Cycles of ^177^Lu-PSMA-617 in Metastatic Castration-Resistant Prostate Cancer with Diffuse Bone Marrow Involvement

**DOI:** 10.3390/cancers13164017

**Published:** 2021-08-10

**Authors:** Daniel Groener, Justus Baumgarten, Sebastian Haefele, Christian Happel, Konrad Klimek, Nicolai Mader, Christina Nguyen Ngoc, Nikolaos Tselis, Felix K. H. Chun, Frank Grünwald, Amir Sabet

**Affiliations:** 1 Department of Nuclear Medicine, University Hospital Frankfurt, Theodor Stern Kai 7, 60590 Frankfurt, Germany; daniel.groener@kgu.de (D.G.); justus.baumgarten@kgu.de (J.B.); sebastian.haefele@kgu.de (S.H.); christian.happel@kgu.de (C.H.); konrad.klimek@kgu.de (K.K.); nicolai.mader@kgu.de (N.M.); christina.nguyenngoc@kgu.de (C.N.N.); frank.gruenwald@kgu.de (F.G.); 2 Department of Radiation Oncology, University Hospital Frankfurt, Theodor Stern Kai 7, 60590 Frankfurt, Germany; nikolaos.tselis@kgu.de; 3 Department of Urology, University Hospital Frankfurt, Theodor Stern Kai 7, 60590 Frankfurt, Germany; felix.chun@kgu.de

**Keywords:** PSMA, ^177^Lu-PSMA-617, diffuse marrow involvement, metastatic castration-resistant prostate cancer

## Abstract

**Simple Summary:**

Metastatic castration-resistant prostate cancer (mCRPC) with extensive spread to the bone marrow is an incurable stage of disease associated with a poor prognosis and a high risk of impaired blood cell formation. Therapeutic options prolonging survival are limited and may result in significant bone marrow toxicity. The concept of radioligand therapy (RLT) in mCRPC is marked by the targeted delivery of radionuclides, such as beta particle emitting ^177^Lutetium (^177^Lu) to the prostate-specific membrane antigen (PSMA), a transmembrane protein frequently present on prostate cancer cells. RLT has yielded promising anti-tumoral activity and excellent tolerability in patients with mCRPC as shown by multiple retrospective series and a growing number of prospective trials. The presented study aims to investigate the role of RLT in mCRPC patients with metastases diffusely involving the bone marrow. Special emphasis is laid on identifying early indicators for a favorable treatment response and potential risk factors for adverse outcomes. The impact of RLT-specific variables, including administered treatment activity, cumulative activity and whole-body absorbed dose is assessed individually.

**Abstract:**

Advanced stage metastatic prostate cancer with extensive bone marrow involvement is associated with a high risk of therapy-induced myelotoxicity and unfavorable outcomes. The role of salvage radioligand therapy (RLT) with ^177^Lu-PSMA-617 in this subset of patients remains to be further elucidated. Forty-five patients with progressive metastatic castration-resistant prostate cancer (mCRPC) and diffuse bone marrow involvement were treated with repeated cycles of RLT after having exhausted standard treatment options. A mean treatment activity of 7.4 ± 1.4 GBq ^177^Lu-PSMA-617 was administered in a median of four treatment cycles (IQR 2-6) and the mean cumulative activity was 32.6 ± 20.1 GBq. After two RLT cycles, ≥50% PSA decline was observed in 25/45 (56%) patients and imaging-based partial remission (PR) was observed in 18/45 (40%) patients. Median imaging-based progression-free survival (PFS) was 6.4 mo (95% CI, 3.0–9.8) and the median overall survival (OS) was 10.2 months (95% CI, 7.2–12.8). The biochemical response translated into a significantly prolonged PFS (12.9 vs. 2.8 mo, *p* < 0.001) and OS (13.5 vs. 6.7 mo, *p* < 0.001). Patients with PR on interim imaging after two cycles had a longer median OS compared to patients with stable or progressive disease (15.5 vs. 7.1 mo, *p* < 0.001). Previous taxane-based chemotherapy (HR 3.21, 95%CI 1.18–8.70, *p* = 0.02) and baseline LDH levels (HR 1.001, 95%CI 1.000–1.001, *p* = 0.04) were inversely associated with OS on a Cox-regression analysis. Grade ≥ 3 hematological decline was observed after 22/201 (11%) cycles with anemia, leukopenia and thrombocytopenia in 15/45 (33%), 6/45 (13%) and 8/45 (18%) patients, respectively. Cumulative treatment activity and absorbed whole-body dose were not correlated with new onset grade ≥ 3 hematotoxicity (*p* = 0.91, *p* = 0.69). No event of grade ≥ 3 chronic kidney disease was observed during RLT or the follow-up. Last line RLT with ^177^Lu-PSMA-617 in mCRPC patients with diffuse bone marrow involvement may thus contribute to prolonged disease control at an acceptable safety profile.

## 1. Introduction

Prostate cancer (PC) is a leading cause for cancer-related mortality in men [1,2]. While localized PC can be successfully addressed by curative treatment, patients progressing to metastatic castration-resistant prostate cancer (mCRPC) are faced with disease-specific morbidity and poor outcomes [3]. As the clinical picture of mCRPC is frequently marked by the emergence of bone metastases [4], symptomatic bone lesions and skeletal-related events (SREs) may ultimately take a considerable toll on patients’ quality of life [5].

Standard therapeutic options prolonging overall survival (OS) in mCRPC are limited. Available systemic treatments include second-generation antiandrogens (enzalutamide and abiraterone) as well as potentially myelotoxic taxane-based chemotherapy (docetaxel and cabazitaxel) and bone-seeking ^223^Ra-dichloride [6,7,8,9,10,11]. In recent years, radioligand therapy (RLT) directed at the type II transmembrane glycoprotein prostate-specific membrane antigen (PSMA) has been increasingly implemented as a treatment option in mCRPC. Small-molecule PSMA ligands labeled with beta-emitting ^177^Lutetium, such as Glu-urea-based ^177^Lu-PSMA-617 and ^177^Lu-DOTAGA-(I-y)fk(Sub-KuE), briefly termed ^177^Lu-PSMA-I&T have yielded promising anti-tumoral activity, clinical benefit and excellent overall tolerability in multiple prospective and retrospective series [12,13,14,15]. Recently published data from VISION (NCT03511664), a large-scale, open label, multicenter phase three trial further underlined the role of RLT in mCRPC [16].

Once metastatic disease diffusely expands to the bone marrow, hematologic reserves may be compromised or become prone to deterioration, potentially precluding patients from existing cytoreductive therapies and eventually putting them at higher risk for disease progression and further performance status decline. So far, these patients have frequently not been considered for radionuclide therapy trials due to unknown safety aspects and the uncertainty of the clinical benefit. The ALSYMPCA trial excluded mCRPC patients with diffuse bone tumor expansion on bone scan imaging from treatment with ^223^Ra-dichloride [6]. Prospective phase two and phase three trials on ^177^Lu-PSMA-617 have also spared this disease phenotype from investigation [12,14,17].

Management of mCRPC with diffuse bone marrow involvement remains an area of uncertainty, since the general findings from RLT cohorts with heterogeneous tumor burden may not be applicable. To date, only few retrospective reports have addressed the role of RLT in this subset of patients [18,19].

Within the treatment routine of RLT, consisting of multiple cycles in 6–8-week intervals, early response assessment may provide a rationale for treatment continuation after the initial RLT cycle and serve as a basis for clinical decision making [20,21]. This question is of special interest in patients with diffuse marrow involvement receiving RLT in a salvage setting, where treatment alternatives are exhausted and toxicity risks must be weighed against maintaining the patients’ best quality of life. Apart from a biochemical response assessment guided by serial prostate-specific antigen (PSA) sampling, additional biomarkers including total alkaline phosphatase (ALP) and lactate dehydrogenase (LDH) are considered possible surrogates for the disease burden in patients with an extensive tumor load [22].

In this study, we seek to investigate the role of RLT in a sizable cohort of patients with widespread diffuse bone marrow involvement. Special emphasis is laid on identifying the contributing factors for favorable treatment responses and the potential risks for adverse outcomes. The impact of RLT-specific variables, including administered treatment activity, cumulative activity and the whole-body absorbed dose is assessed individually.

## 2. Materials and Methods

### 2.1. Patients

A total of 45 patients received salvage ^177^Lu-PSMA-617 in this retrospective single-center series. The inclusion criteria mandated that patients had progressive mCRPC with diffuse bone marrow involvement extending beyond the axial skeleton and no remaining standard treatment alternatives. Sufficient PSMA-expression in the target lesions was pre-ascertained by ^68^Ga-PSMA-11 PET/CT imaging. Diffuse bone marrow involvement was defined as set out by the Prostate Cancer Molecular Imaging Standardized Evaluation (PROMISE, M1b, dmi) [23]. Indications were confirmed by an interdisciplinary team including board-certified nuclear medicine physicians, urologists, radiation oncologists, pathologists and oncologists. Further requirements for treatment initiation included an estimated glomerular filtration rate (eGFR (based on the Chronic Kidney Disease Epidemiology Collaboration equation)) of >30 mL/min/1.73 m^2^, hemoglobin ≥ 8.0 g/dL, white blood cells (WBC) ≥ 2.00 × 10^9^/L and platelets ≥ 75 × 10^9^/L. The production and administration of ^177^Lu-PSMA-617 were performed in accordance with legal regulations set out in the German Drug Registration and Administration Act (AMG § 13 2b). All patients gave written informed consent prior to each therapy cycle and retrospective data analysis was approved by the local ethics committee (approval number: 310/18).

### 2.2. Radiolabeling and Administration

The radiolabeling of PSMA-617 with ^177^LuCl_3_ was carried out as has been described in detail previously [24,25,26]. The automated synthesis of ^177^Lu-PSMA-617 was performed on the Gaia/Luna GMP automated radiosynthesizer (Elysia-raytest GmbH, Straubenhardt, Germany) with sterile, single-use cassettes and reagent kits (ABX GmbH, Dresden, Germany) using the ^177^Lu (EndolucinBeta, ITM, Garching, Germany) delivery vial as the reaction vessel. For labeling, 9 µg PSMA-617 (ABX GmbH, Dresden, Germany) per GBq was used. During the automated process, the aqueous PSMA-617 stock solution (1 mg/mL) in a 1000 µL buffer (gentisic acid/sodium ascorbate/HCl) was transferred to the ^177^Lu vessel and subsequently heated to 95 °C for 30 min. After cooling and dilution with 0.9% NaCl, the product was passed through a sterile filter and further diluted with 0.9% NaCl to a volume of approximately 10 mL. Quality control was overseen by experienced radiochemists and physicians with respective training in the field. ^177^Lu-PSMA-617 was administered by slow intravenous injection over 30–60 s, preceded and followed by 1000 mL of saline infusion. All therapies were performed as in-patient procedures at the nuclear medicine therapy ward.

### 2.3. Toxicity Assessment

Repeat sampling of the hematological parameters (hemoglobin, white blood cells and platelets), biomarkers (PSA, ALP, LDH) and renal function based on the estimated glomerular filtration rate (eGFR) were undertaken at the baseline, prior to each therapy cycle, 2 to 4 weeks after each cycle and in 6 to 12 week intervals throughout the follow-up. The performance level based on the Eastern Cooperative Oncology Group (ECOG) status and pain levels quantified through a visual analog scale (VAS) ranging from 0 to 10 were assessed at the baseline and at each treatment cycle. The severity of adverse events was graded based on Common Terminology Criteria for Adverse Events (CTCAE), version 5.0, with grade ≥ 3 toxicities being termed significant.

### 2.4. Response Assessment

Biochemical and imaging responses were analyzed according to the criteria set out by the Prostate Cancer Working Group 3 (PCWG3) [27]. The PSA response was defined as a ≥50% decline from treatment initiation, progression was defined as ≥25% increase exceeding 2 ng/mL, confirmed by a second measurement ≥ 3 weeks apart and values between these limits were considered stable. Initial PSA progression after one cycle of RLT was not considered a discontinuation criterion for RLT in patients otherwise clinically benefiting. Based on previous reports, total ALP decline ≥ 30% was classified a significant ALP response [22]. Overall survival (OS) was defined as the time from RLT initiation (date of first administration) to death from any cause. ^68^Ga-PSMA-11 PET/CT imaging was performed after a minimum of 2 RLT cycles and afterwards every 2 cycles. The assessment of the PSMA-imaging response was based on a recently met consensus [28]: partial response (PR) was defined as a reduction in uptake/tumor volume by >30%; stable disease (SD) was defined as a change of uptake/tumor volume ≤ 30% without evidence of new lesions; progressive disease (PD) was defined as the appearance of ≥2 new lesions, an increase in uptake/tumor volume by >30% or the peripheral expansion of diffuse bone marrow involvement. Progression-free survival (PFS) was defined as the time from RLT initiation to imaging-based progression or death, whichever occurred first.

### 2.5. Whole-Body Dosimetry

Intra-therapeutic whole-body dosimetry was performed by sequential measurements of the remaining activity within the body using a calibrated gamma probe (2” × 2” NaI(Tl) detector with multichannel analyzer-scintiSPECT, SCINTRONIX). The first measurement was performed directly after the intravenous administration of ^177^Lu-PSMA-617 prior to bladder voiding and served as calibration for all subsequent measurements. The following measurements were performed 2, 4, 20, 24, 28, 44, 48, 52, 68, 72, 76 and 92 h after administration. Residual activity in the patient was calculated using the background compensated geometric mean of the ventral and dorsal count rate related to the individual background compensated calibration factor. Patients were measured in the ventral and dorsal position standing 6 m distance from the gamma probe to avoid dead time effects. An energy window of 208 keV ±20% was used. The whole body dose was determined by approximating the time activity curve using a bi-exponential fit (Solver MS Excel 2010) and estimating the number of decays in the body by calculating the area under this curve. A mean deposited energy of 2.34 × 10^−14^ J per decay was used and related to the body weight of the patient [29].

### 2.6. Statistical Analysis

Results are presented as the median with the interquartile range (IQR) and mean ± standard deviation for continuous variables. Categorical variables are reported as frequencies with respective percentages. The paired Student’s *t*-test was used to compare intraindividual changes in continuous biochemical parameters. The toxicity analysis was carried out per patient (patient-based) and per cycle (cycle-based) as indicated. Significant hematologic toxicity was defined as an increase in toxicity to grade 3 or higher throughout the course of RLT and transformed into a dichotomized variable. The association of continuous variables was analyzed using a parametric correlation (Pearson’s correlation coefficient denoted with r) and a non-parametric rank correlation (Spearman’s correlation coefficient denoted with r_s_) was performed in cases with categorical parameters. Progression-free (PFS) and overall survival (OS) were calculated based on the Kaplan–Meier method (log-rank testing). A univariate analysis based on a log-rank test was performed for baseline factors of interest. Variables showing a relevant tendency to impact the outcome (*p* < 0.10) were incorporated into a multivariable cox regression model. Statistical analyses were performed with SPSS (version 27.0, IBM, Armonk, NY, USA) and GraphPad Prism (version 9.1.1, GraphPad Software, San Diego, CA, USA) was used to plot graphs. All tests were two-sided with *p*-values < 0.05 denominating statistical significance.

## 3. Results

Forty-five consecutive patients with progressive mCRPC (median age 71 (IQR 68–76) years) presenting with diffuse bone marrow involvement underwent RLT at our institution. Previous treatments consisted of multiple lines of systemic treatment, including abiraterone and/or enzalutamide in 40/45 (89%), at least one line of taxane-based chemotherapy in 36/45 (80%) and ^223^Ra-dichloride in 12/45 (27%) patients. Patient characteristics at the baseline are further detailed in Table 1. A total of 201 cycles of ^177^Lu-PSMA-617 were administered with a mean treatment activity of 7.4 ± 1.4 GBq applied in median of four (IQR 2–6) treatment cycles per patient. All patients received a minimum of two treatment RLT cycles, given at intervals of 4–8 weeks and reaching a mean cumulative activity of 32.6 ± 20.1 GBq.

### 3.1. Response

**Figure 1:**
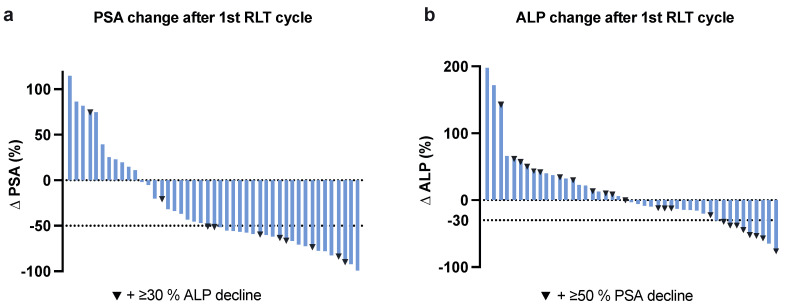
Waterfall plots indicating (**a**) PSA and (**b**) ALP response after first RLT cycle.

**Figure 2:**
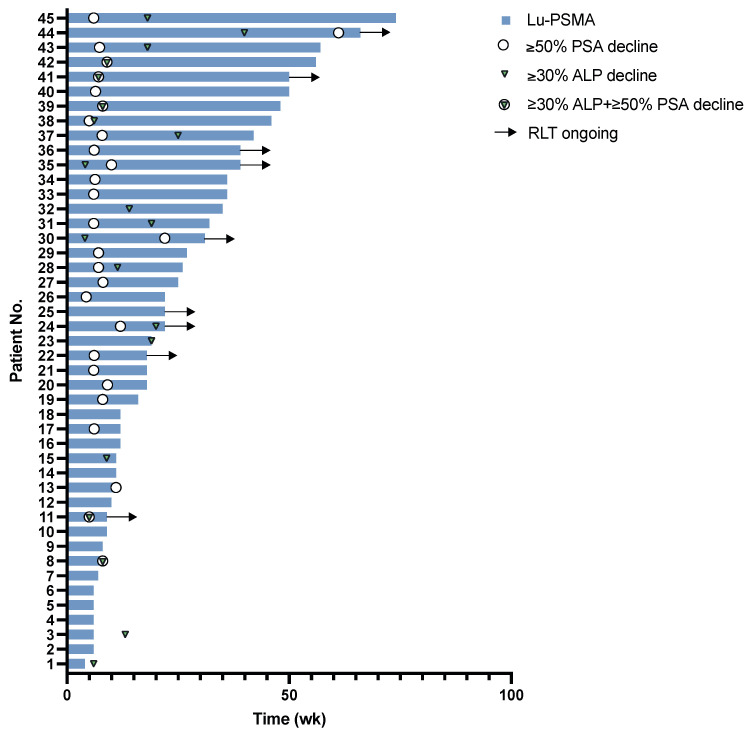
Swimmer plot with response events throughout RLT.

**Figure 3:**
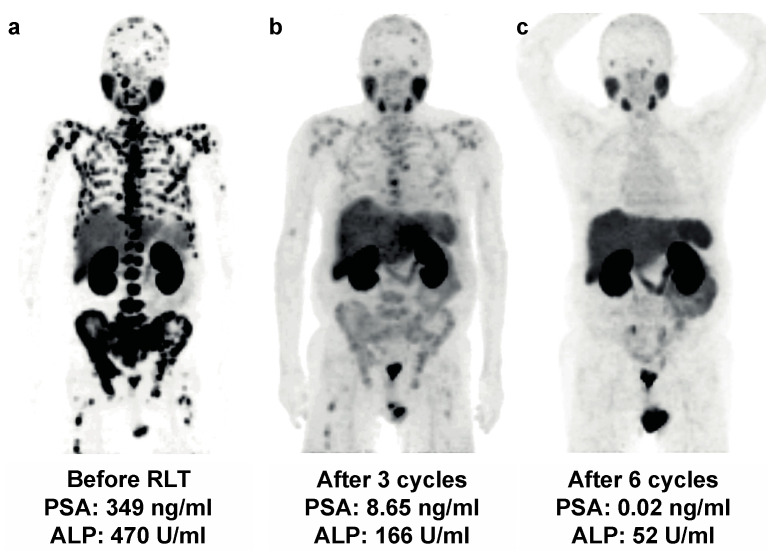
Maximum intensity projections of ^68^Ga-PSMA-11 PET/CT imaging in a 69-year-old patient at the baseline (**a**), upon interim staging (**b**), with excellent treatment response after 6 cycles of ^177^Lu-PSMA-617 after 46.3 GBq of cumulative treatment activity (**c**).

After the first RLT cycle, 24/45 (53%) patients showed ≥50% PSA decline, 14/45 (31%) patients remained stable and 7/45 (16%) patients had ≥25% PSA progression. Of seven patients with progressive PSA values ≥ 25% after one cycle, three (43%) ultimately had stable disease and one (14%) experienced a partial response. The course of total ALP was analyzed, yielding ≥ 30% ALP decline after one RLT cycle in 10/45 (22%) patients. Of the 24 patients with initial ≥ 50% PSA decline 8/24 (33%) had concomitant ≥ 30% ALP decline after one cycle and an additional five PSA-responders developed ≥ 30% ALP decline throughout the course of RLT. Waterfall and swimmer plots for PSA and ALP responses are shown in Figure 1 and Figure 2. An interim analysis was performed after a minimum of two treatment cycles. Here, the biochemical response consisted of ≥50% PSA decline in 25/45 (56%) patients, stable values in 11/45 (24%) patients, and ≥25% PSA progression in 10/45 (22%). The imaging assessment showed 18/45 (40%) of all treated patients had a partial response (PR), 20/45 (44%) remained stable (SD) and 7/45 (16%) had progressive disease (PD). An example of the treatment response on ^68^Ga-PSMA-11 PET/CT imaging upon the interim and the follow-up are provided in Figure 3. Among 24 patients clinically impacted by pain at the baseline (defined as ≥4 pain level on a VAS assessment), 16/24 (67%) reported a significant improvement in the overall pain level (≥2 decline) throughout RLT. An improvement in the performance status level (ECOG level) was documented in nine patients.

### 3.2. Survival

**Figure 4:**
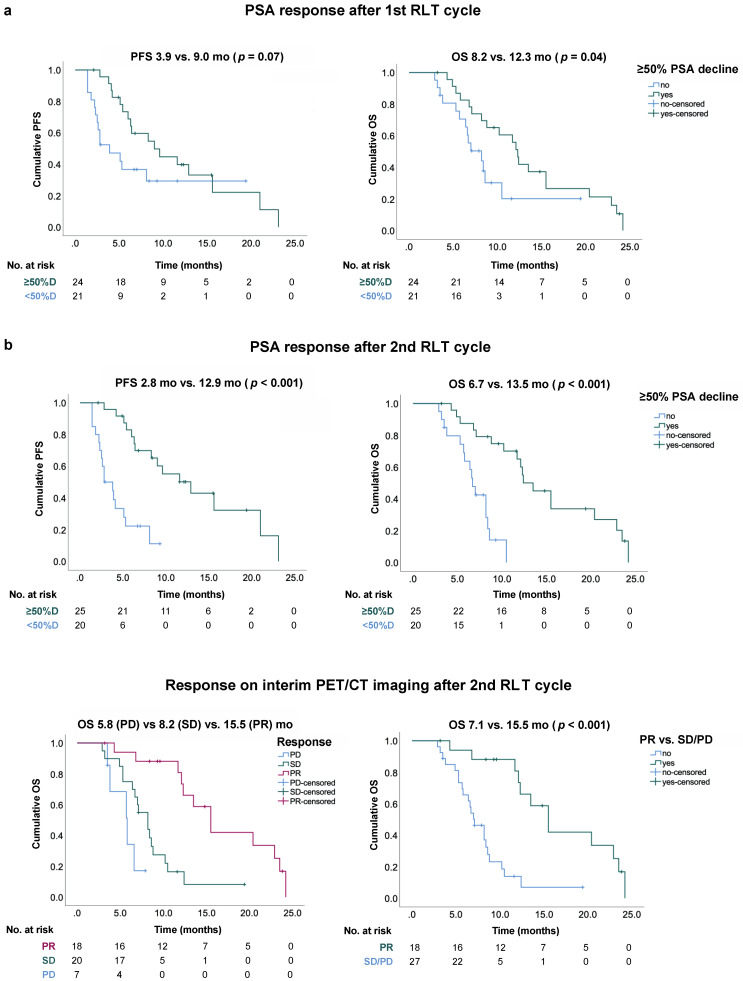
Kaplan–Meier curves (**a**) after 1st and (**b**) 2nd RLT cycle; PFS: progression-free survival, OS: overall survival, PR: partial response, SD: stable disease, PD: progressive disease.

The mean follow-up was 11.2 ± 8.5 months. By the time of the analysis, 34/45 (76%) patients were deceased. The median overall survival (OS) for the entire study cohort was 10.2 mo (95% CI, 7.3–13.1) and the median imaging-based progression-free survival (PFS) was 6.4 mo (95% CI, 3.0–9.8). Early ≥50% PSA decline after one cycle resulted in a prolonged median OS (12.3 vs. 8.2 mo, *p* = 0.04). Moreover, patients with early ≥30% ALP decline had a prolonged median OS (15.5 vs. 8.2 mo, *p* = 0.01). After two RLT cycles, both biochemical and imaging-based responses were significantly associated with a better outcome. Patients with PR on the imaging-based assessment had a longer median OS (15.5 mo, 95% CI 12.3–18.7) compared to SD (8.2, 95% CI 6.9–10.4, *p* < 0.001) or PD (5.8 mo, 95% CI 3.6–8.0, *p* < 0.001). PSA decline ≥ 50% after two cycles was associated with both a longer median PFS (12.9 vs. 2.8 mo, *p* < 0.001) and OS (13.5 vs. 6.7 mo, *p* < 0.001). The Kaplan–Meier plots are shown in Figure 4.

The Cox-regression analysis for the various baseline factors as to their contribution to OS is detailed in Table 2. On the univariate regression, LDH levels at the baseline and prior to the taxane-based chemotherapy were associated with an adverse impact on OS (*p* = 0.02, *p* = 0.02). Previous treatment with ^223^Ra-dichlorid showed no significant contribution to OS in subsequent RLT cycles (*p* = 0.90). Hepatic metastases showed a tendency towards a poorer OS (*p* = 0.07). Upon multivariable regression, both taxane-based chemotherapy (HR 3.21, 95% CI 1.18–8.70, *p* = 0.02) and the baseline LDH levels (HR 1.001, 95% CI 1.000–1.001, *p* = 0.04) remained adverse prognosticators of OS.

### 3.3. Safety

Prior to the RLT cycles, 41/45 (91%) patients had low grade anemia (25 grade 1, 16 grade 2), 6/45 (13%) had leukopenia (2 grade 1, 4 grade 2) and 9/45 (20%) had thrombocytopenia (8 grade 1, 1 grade 2). Three patients with hemoglobin levels slightly below the inclusion threshold (7.7, 7.6 and 7.6 g/dL) were treated given the lack of therapeutic alternatives. Hematologic parameters showed a slight but significant absolute decline through the course of RLT. The median hemoglobin decreased from 10.2 (IQR 8.8–11.3) g/dL at the baseline to 8.3 (IQR 7.6–9.6) g/dL at the maximum level of deterioration (*p* < 0.001), the median WBC counts shifted from 6.20 (IQR 4.85–7.69) × 10^9^/L to 3.20 (IQR 2.55–3.91) × 10^9^/L (*p* < 0.001), and the thrombocytes decreased from 215 (IQR 148–289) × 10^9^/L to 91 (IQR 56–137) × 10^9^/L (*p* < 0.001).

Significant hematologic adverse events (grade ≥ 3) during RLT occurred in 18/45 (40%) patients, with new onset anemia in 15/45 (33%), leukopenia in 6/45 (13%) and thrombocytopenia in 8/45 (18%), as summarized in Table 3. The median cumulative activity prior to grade ≥ 3 toxicity was 22.3 (IQR 9.7–34.4) GBq. Of the 18 patients affected by significant hematologic toxicity, 11/18 (69%) had presented with initial grade 2 cytopenia, 16/18 (89%) had a history of taxane-based chemotherapy and 5/18 (28%) had undergone ^223^Ra-dichloride prior to RLT. Of the 201 cycles administered, 22/201 (11%) were subject to subsequent grade ≥ 3 toxicity, which occurred within a median of 6 weeks of the preceding treatment cycle. Cumulative treatment activity was not correlated with the occurrence of grade ≥ 3 toxicity either per patient or per cycle (*p* = 0.91, *p* = 0.57).

No case of grade ≥ 3 chronic kidney disease (based on eGFR) was observed during RLT and throughout the follow-up period. Six patients had new onset grade 2 chronic kidney disease, which in 3/6 (50%) patients reverted to grade ≤ 1 on follow-up, the remaining 3/6 (50%) remained at stable eGFR levels. One patient developed acute renal failure by obstruction due to a lymph nodal tumor burden, which was successfully treated by a double-J stent placement allowing for five additional cycles of RLT at normal eGFR levels. New onset skeletal-related events (SREs) were recorded in two patients; one developed a pathological bone fracture to the thoracic skeleton and the second showed a stable vertebral fracture. All SREs could be managed conservatively.

### 3.4. Patients with Significant Toxicity

Of the 18 patients with grade ≥ 3 hematologic toxicities, two spontaneously recovered to lower levels (grade ≤ 2) within 4 to 10 weeks. Fourteen (78%) patients with significant myelosuppression received transfusion therapy, thirteen of which were transfused with packed red blood cells and three received platelet concentrates. Three (17%) patients could receive additional cycles of RLT either after spontaneous recovery or blood transfusion. Cytopenia was thus successfully managed in 11 patients. Four patients who experienced significant disease progression following their last cycle died within 4 to 10 weeks after RLT and two patients were lost prior to the follow-up. Of the three study patients included with grade 3 anemia upon treatment initiation, two spontaneously recovered to grade 2 after responding to RLT and one received packed red blood cells throughout the course of RLT and remained at stable grade 2 hemoglobin levels prior to discontinuing RLT due to disease progression. 

### 3.5. Whole-Body Dosimetry

The median whole-body absorbed dose was 0.53 (IQR 0.34–0.93) Gy per treatment cycle, corresponding to 0.076 (IQR 0.049–0.128) Gy/GBq per activity unit. A significant correlation of PSA levels and whole-body absorbed dose per activity unit was observed at the baseline (r = 0.46, *p* < 0.001) and throughout all treatment cycles administered (r = 0.39, *p* = 0.007). While the whole-body absorbed dose per cycle was slightly correlated with a subsequent decline in leukocyte (r = 0.16, *p* = 0.03) and thrombocyte counts (r = 0.23, *p* = 0.002), there was no significant association with occurrence of new onset grade ≥ 3 hematotoxicity (r_s_ = 0.03, 0.69).

## 4. Discussion

In patients with mCRPC and diffuse bone marrow involvement, RLT with ^177^Lu-PSMA-617 achieved high therapeutic efficacy and favorable tolerability in a salvage setting after extensive prior therapy and the exhaustion of standard treatment lines.

Biochemical response defined as ≥50% PSA decline was observed in 25/45 (56%) patients and 18/45 (40%) showed imaging-based PR after two RLT cycles. This compares favorably to published prospective data form patient cohorts with various extents of tumor burden, which reported PSA response rates in the range of 57–64%, and imaging-based PR/CR in 43% after 12 weeks of RLT [14,30]. Our findings suggest that even in the presence of extensive bone tumor load, the response to RLT with beta-emitting ^177^Lutetium is widely maintained.

The median OS in our study was 10.2 mo (95% CI, 7.2–12.8). In comparison, Hofman et al. reported a median 13.5 mo OS in their phase two ^177^Lu-PSMA-617 trial [12]. In the international phase three study VISION (NCT03511664) a median OS of 15.3 mo was reached in patients treated with ^177^Lu-PSMA-617 in addition to the best supportive care [31]. The expected longer OS in these prospective studies reasonably reflects the lower baseline tumor burden in their patient populations through the initial exclusion of diffuse bone metastases by the protocol. Only few retrospective series have addressed the feasibility of RLT in patients with diffuse bone marrow involvement. Gafita et al. analyzed 43 patients receiving a total of 154 cycles [18]. The reported median OS following the landmark assessment 12 weeks after treatment initiation was 11.6 mo and the median time to PSA progression was 4.8 mo. Within the study, 5/43 (12%) patients received up to 11 additional RLT cycles in a rechallenge concept after the initial response to RLT [20]. This may account for the somewhat longer OS compared to our study, which was restricted to a last-line salvage setting without subsequent systemic therapeutics. A multicenter study conducted by Ahmadzadehfar et al. (WARMTH-617 trial) yielded median OS of 8.2 mo in 83 heavily pretreated patients with diffuse osseous metastases. The shorter OS compared to our study could be a result of the higher portion of patients with liver metastases in their group [19]: in the WARMTH-617 trial 27/83 (33%) patients with diffuse bone marrow involvement and liver metastases had a worse OS compared to patients without a hepatic tumor load (5.7 vs. 9.0 mo, *p* < 0.001). In our cohort a fraction of 5/45 (11%) patients presenting with hepatic metastases also had a shorter median OS (5.3 vs. 10.5 mo), though this finding did not meet statistical significance. Several previous studies support the hypothesis that concomitant hepatic metastases have an adverse impact on outcome of RLT [13,15,32].

The imaging-based PFS in our cohort was 6.4 mo (95% CI, 3.0–9.8), which appears only slightly inferior compared to reports on PFS from cohorts with heterogeneous tumor burden [26,33]. To the best of our knowledge, imaging-based PFS has so far not been subject to analysis in patients presenting with diffuse bone marrow involvement upon RLT initiation.

To refine therapeutic reasoning in RLT, early denominators of treatment efficacy are highly desired. Various predictive factors for both response and outcome have been put forth, mainly based on retrospective evidence from heterogeneous patient groups [34]. 

Total ALP is correlated with the tumor burden in mCRPC and may play a distinct role as a biomarker in patients with a high bone tumor load [35]. Several studies have investigated ALP at the baseline as a predictive factor, mostly by establishing cut-off values for adverse outcome [15,36,37]. Barber et al. found ALP values exceeding 220 U/mL at baseline to be associated with poorer PFS and OS [38]. In our study patients presented with a higher tumor burden at the baseline, resulting in a median baseline ALP considerably exceeding the upper reference limit. Within this elevated ALP range, we observed no correlation of outcome with ALP levels. Thus, baseline ALP may be more suitable to stratify tumor load in heterogeneous cohorts; it appears to have limited discriminatory value in patients with high volume disease. Upon Cox-regression, baseline LDH was significantly associated with decreased OS. This is in line with a post hoc analysis from ALSYMPCA and published retrospective RLT trials, where higher LDH levels at baseline were prognostic for adverse outcomes [13,22,37,39,40].

In our cohort, previous taxane-based chemotherapy was associated with decreased OS. This result must be interpreted with caution, given the limited sample size of nine patients without previous taxanes. As reported by Ahmadzadehfar et al. in their multicenter study, taxane-pretreated patients with diffuse bone involvement had a tendency towards a worse OS (7.8 vs. 11.0 mo) [19].

Biochemical responses to early cycles of RLT may anticipate the later course of treatment and outcome. In addition to response assessment after ≥12 weeks as advocated by PCWG3 response criteria [27], cycle-based routine in RLT provides further timepoints for analyses. In our study, patients with ≥50% PSA decline after the first RLT cycle had longer OS. Though this finding may be useful for early clinical decision making, it must be noted that a significant portion of patients (57%) with PSA progression ≥ 25% after one cycle later responded to RLT with SD or PR. Thus, in our cohort, early progression was frequently not indicative of subsequent disease course. Interim response on PSMA-imaging after two RLT cycles may be more suitable to prognosticate subsequent outcome. Here, patients with PR had significantly prolonged OS compared with SD or PD, which is compatible with recent studies from heterogeneous cohorts emphasizing the role of interim imaging after two to three RLT cycles [41,42].

In radionuclide therapy, blood-driven recirculating ß-irradiation and scatter radiation from bone metastases can induce or aggravate myelosuppression [43,44]. In our cohort, significant (grade ≥ 3) hematologic toxicity occurred after 10.9% of all treatment cycles, with anemia in 33.3%, leukopenia 13.3% and thrombocytopenia in 17.7% of all patients treated. Delimiting pre-existing cytopenia worsened by disease progression from true therapy-emergent toxicity remains challenging, since both phenomena may occur simultaneously. For a conservative estimate and comparability with other studies, we included all new onset grade ≥ 3 toxicities in our analysis, regardless of disease progression being the most likely cause in several cases. To approximate the natural course of mCRPC in patients with bone-dominant tumor load, the placebo arm of the ALSYMPCA trial may provide a useful comparison [6]. In this group of 301 mCRPC patients with bone-dominant disease, new onset grade ≥ 3 anemia, neutropenia and thrombocytopenia occurred in 13%, 1% and 3% of patients [45].

Since the introduction of ^177^Lu-labeled radioligands targeting PSMA, multiple studies have included descriptive analyses of hematologic adverse events, overall reporting moderate rates of grade ≥ 3 toxicities in the range of 8–24% for anemia, 0–8% for leukopenia and 1–11% for thrombocytopenia [14,15,38,46]. Two aforementioned studies have assessed hematological safety in selected patient cohorts with diffuse marrow involvement [18,19]. Gafita et al. analyzed 154 cycles of RLT in 43 patients, yielding grade ≥ 3 anemia, neutropenia and thrombocytopenia, in 22.5%, 7.5%, and 25.0%, respectively. The multicenter analysis with 83 patients conducted by Ahmadzadehfar et al. found grade ≥ 3 anemia, leukopenia and thrombocytopenia in 24.3%, 0% and 6.6% of patients [19]. Our results slightly exceed the above findings, which may be partly attributed to the fact that our cohort was treated in a salvage setting, with arguably further disease progression to the bone marrow at baseline. The impact of cumulative activity or whole-body absorbed dose on hematotoxicity in patients with diffuse-bone marrow involvement have so far not been subject to analysis. In our study, higher absorbed doses to the whole body were associated with decreasing leukocyte and thrombocyte counts but did not lead to excess grade ≥ 3 hematologic toxicity. Moreover, cumulative activity administered to patients did not contribute to higher rates of grade ≥ 3 hematoxicity. The latter findings are a likely result of individual dose de-escalation and must be interpreted carefully due to potential bias.

With the exception of one case of acute renal deterioration due to reversible obstruction, significant renal impairment was not observed during RLT or subsequent follow-up. It remains to be investigated, whether extensive tumor load in diffuse bone marrow involvement is inversely correlated with renal toxicity through predominant concentration of treatment activity in tumor sites, as previously suggested [47].

Relevant limitations of this study are its population size and the retrospective nature which inevitably impacts the strength of conclusions drawn. Comparison with other retrospective cohorts should be interpreted with caution, while prospective validation in larger cohorts is yet to be conducted.

## 5. Conclusions

Our findings suggest that repeated cycles of RLT with ^177^Lu-PSMA-617 can be carried out at an acceptable safety profile in mCRPC patients with diffuse bone marrow involvement. RLT may provide a suitable strategy for prolonged disease control with substantial clinical benefit. After a minimum of two cycles, treatment response translated into significantly longer median OS. Further studies should be carried out to investigate the role of individualized RLT concepts in this advanced stage setting.

## Figures and Tables

**Table 1:** Baseline characteristics.

Variable	All Patients (*n* = 45)
Age	71 (68–76)
PSA (µg/L)	551 (339–1280)
ALP (U/L)	373 (153–598)
LDH (U/L)	297 (239–440)
Hemoglobin (g/L)	10.4 (8.8–11.3)
White blood cells (10^9^/L)	6.2 (4.9–7.7)
Platelets (10^9^/L)	215 (148–289)
eGFR (mL/min/1.73 m^2^)	88.2 (70–96.6)
Gleason score *	
<8	12 (32)
≥8	25 (68)
**ECOG performance status**	
1	20 (44)
2–3	25 (56)
**Pain**	
No pain/controlled pain	21 (47)
Pain	24 (53)
**Sites of metastases**	
Bone	45 (100)
Lymph nodes	26 (58)
Visceral	10 (22)
- hepatic	5 (11)
- pulmonary	3 (7)
**Prior systemic therapies for mCRPC**	
Abiraterone	34 (76)
Enzalutamide	32 (71)
^223^Ra-dichloride	12 (27)
Docetaxel	36 (80)
Cabazitaxel	20 (44)
Other chemotherapies **	2 (4)
**External beam radiotherapy**	
Primary site	17 (38)
Metastatic site	22 (49)

Data presented as median with interquartile range (IQR) or *n* (%), *: for *n* = 37 available patients, PSA: prostate-specific antigen, ALP: total alkaline phosphatase LDH: lactate dehydrogenase, eGFR: estimated glomerular filtration rate, ECOG: Eastern Cooperative Oncology Group, ** Cisplatin, 5-FU. Bold: hierarchy of baseline characteristcs.

**Table 2:** Univariate and multivariable Cox-regression.

Risk Factor			Univariate			Multivariable	
	*n*	(%)	*p*		HR	95% CI	*p*
Gleason score *							
<8	12	32					
≥8	25	68	0.52				
**ECOG status at baseline**							
1	20	44					
2–3	25	56	0.47				
**Visceral metastases at baseline**							
- any	10	22	0.23				
- hepatic	5	11	0.07		1.82	(0.54–6.17)	0.34
**Previous mCRPC therapies**							
- Taxane-based chemotherapy	36	80	0.02		3.21	(1.18–8.70)	0.02
- ^223^Ra-dichloride	12	27	0.90				
- Palliative radiotherapy	22	49	0.41				
**Continuous baseline variables**							
PSA (per ng/mL)			0.48				
ALP (per unit)			0.43				
LDH (per unit)			0.02		1.001	(1.000–1.001)	0.04

*: for *n* = 37 available patients, HR: hazards ratio, ECOG: Eastern Cooperative Oncology Group, PSA: prostate-specific antigen, ALP: total alkaline phosphatase, LDH: lactate dehydrogenase. Bold: hierarchy of baseline characteristcs.

**Table 3:** Baseline and intra-/post-therapeutic hematologic toxicity grades based on CTCAE v5.0.

Toxicity	Baseline (%)					Intra-/Posttherapeutic (%)			
	Grade 1	Grade 2	Grade 3	Grade 4		Grade 1	Grade 2	Grade 3	Grade 4
Anemia	25 (56)	16 (36)	3 (7)	0 (0)		10 (22)	18 (40)	17 (38)	0 (0)
Leukopenia	2 (4)	4 (9)	0 (0)	0 (0)		17 (38)	11 (24)	6 (13)	0 (0)
Thrombocytopenia	8 (18)	1 (2)	0 (0)	0 (0)		18 (40)	10 (22)	6 (13)	2 (4)
Chronic kidney disease (eGFR)	19 (42)	7 (16)	0 (0)	0 (0)		21 (47)	13 (29)	0 (0)	0 (0)

eGFR: estimated glomerular filtration rate.

## Data Availability

The datasets analyzed and/or analyzed during the current study are available from the corresponding author on reasonable request.
